# Solvation Engineering via Fluorosurfactant Additive Toward Boosted Lithium-Ion Thermoelectrochemical Cells

**DOI:** 10.1007/s40820-023-01292-2

**Published:** 2024-01-04

**Authors:** Yinghong Xu, Zhiwei Li, Langyuan Wu, Hui Dou, Xiaogang Zhang

**Affiliations:** Jiangsu Key Laboratory of Electrochemical Energy Storage Technologies, College of Material Science and Technology, Nanjing University of Aeronautics and Astronautics, Nanjing, 211106 People’s Republic of China

**Keywords:** Solvation engineering, Fluorosurfactant, Ionic thermoelectric, Lithium-ion thermoelectrochemical cell, Low-grade heat

## Abstract

Lithium-ion thermoelectrochemical cell (LTEC), featuring simultaneous energy conversion and storage, has emerged as promising candidate for low-grade heat harvesting. However, relatively poor thermosensitivity and heat-to-current behavior limit the application of LTECs using LiPF_6_ electrolyte. Introducing additives into bulk electrolyte is a reasonable strategy to solve such problem by modifying the solvation structure of electrolyte ions. In this work, we develop a dual-salt electrolyte with fluorosurfactant (FS) additive to achieve high thermopower and durability of LTECs during the conversion of low-grade heat into electricity. The addition of FS induces a unique Li^+^ solvation with the aggregated double anions through a crowded electrolyte environment, resulting in an enhanced mobility kinetics of Li^+^ as well as boosted thermoelectrochemical performances. By coupling optimized electrolyte with graphite electrode, a high thermopower of 13.8 mV K^−1^ and a normalized output power density of 3.99 mW m^–2^ K^–2^ as well as an outstanding output energy density of 607.96 J m^−2^ can be obtained. These results demonstrate that the optimization of electrolyte by regulating solvation structure will inject new vitality into the construction of thermoelectrochemical devices with attractive properties.

**Supplementary Information:**

The online version contains supplementary material available at 10.1007/s40820-023-01292-2.

## Highlights

- Solvation engineering toward dual-salt electrolyte by fluorosurfactant additive is proposed, which implements the stable interface of electrode/electrolyte coupled with fast ion thermodiffusion, improving the durability and capability of lithium-ion thermoelectrochemical cell.
- By combining the optimized electrolyte with functional electrodes, as-constructed device exhibits high Seebeck coefficient and energy density based on hybrid mechanisms, which can be extended as self-power supply for smart electronics.

**Supplementary Information:**

The online version contains supplementary material available at 10.1007/s40820-023-01292-2.

## Graphical abstract

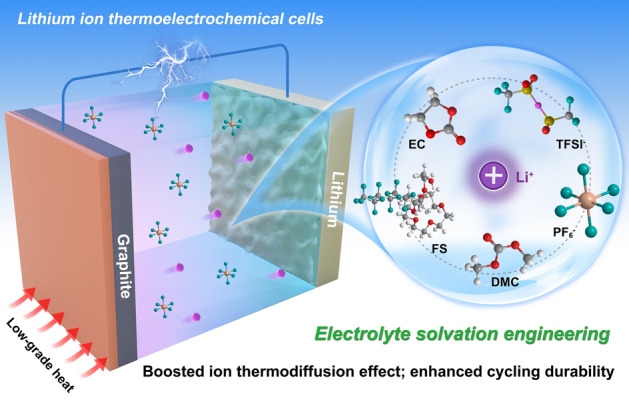

## Introduction

Low-grade heat is ubiquitous in industry, environment and human body; however, most of them are directly discarded without being properly exploited. In order to meet energy conservation and pollution reduction targets with reducing carbon emissions, the sustainable and cost-effective technology of heat to electricity should be considered in priority [1]. Thermoelectric devices, as one of attractive candidates, can integrate thermal energy harvesting, conversion, and storage [2, 3]. The traditional solid-state thermoelectrics (s-TEs) still suffer from the insufficient thermopower with only about 200 μV K^−1^ [4, 5]. Owing to the strong interdependence among thermopower, electrical conductivity and thermal conductivity, it is encountering difficulties to further boost the thermopower of TEs. Moreover, the complicated integration and use of rare element-based materials increase economic costs. In regard of this, the ionic thermoelectrics (i-TEs) based on the temperature-dependent redox reactions at two electrodes (thermogalvanic effect) and ionic thermodiffusion (thermodiffusion effect or Soret effect) under temperature gradient are attracting a growing interest, which demonstrate high thermopower (on the order of mV K^−1^) and satisfying potential in practical applications [6, 7].

Based on the Onsager’s theory, the output of i-TEs highly depends on the thermodynamics and kinetics [8, 9]. From the thermodynamics, the thermovoltage is generated to counterbalance the driving force induced by the applied temperature gradient [10]. And the competence to generate that counterforce at a certain temperature difference can be described by thermopower (*S*_*i*_) [11]:

1 $${S}_{i}=\frac{\Delta V}{\Delta T}=\frac{\Delta S}{nF}$$

where Δ*S*, *n* and *F* are entropy change, the number of electrons transferred and Faraday constant, respectively. Different from thermovoltage generated by the mobility of hole/electron in s-TEs, i-TEs deliver higher thermovoltage value owing to the entropy change of systems by involved redox reactions [12]. Notably, the research regarding i-TEs is still in early stage. There is a key challenge for i-TEs with impressive characteristics like high output power density and energy density as well as long-term cyclic stability.

Since silver iodide was first reported as a feasible electrolyte for i-TEs, researchers have made great efforts in designing better electrodes and electrolytes [1, 13, 14]. Recently, electrode materials with good charge transfer kinetics such as conducting polymers and MXene-based materials have been well studied [15–18]. However, the compatibility between electrodes and electrolytes still is the important project for i-TEs to achieve satisfactory performances. Among the reported electrolytes for i-TEs, aqueous electrolyte has been widely investigated by virtue of fast ion diffusion and good compatibility for redox couples. For instance, the competitive thermoelectric performance of i-TEs can be achieved after introducing redox ion pairs such as Fe^2+^/Fe^3+^, Fe(CN)_6_^3 –^/Fe(CN)_6_^4–^ and I^−^/I_3_^−^ into aqueous electrolytes [19–25]. Moreover, the thermopower can be further improved by adjusting the coordination environment of redox ions [24, 26, 27]. Despite these advances, instantaneous and continuous output energy of such i-TEs using aqueous electrolyte during low-grade heat conversion is still unsatisfactory, which is mainly limited by the aqueous electrolyte properties and ions adsorption/desorption or redox-type reactions on the electrode surfaces [12]. Thus, the design and exploration of functional electrolytes with high activity and large size difference would contribute good thermoelectrochemical performances. In addition, how to realize the integration of energy conversion and storage is still the important project in heat-to-current applications. Lithium-ion batteries (LIBs) have been regarded as typical devices and attract enormous interest in energy storage due to their inherent merits such as high energy and satisfactory stability [28]. The combination of LIBs and i-TEs gives rise to a further exploration of promising candidates for thermoelectrochemical conversion. In our previous work, we developed the lithium-ion thermoelectrochemical cells (LTECs) and demonstrated the synergistic effect among the thermogalvanic effect of electrode and the thermodiffusion of ions in electrolyte [29]. It is worth noting that there are some challenges for the development of LTECs in investigating the relationship between the performances and the solvation of electrolyte ions.

**Fig. 1:**
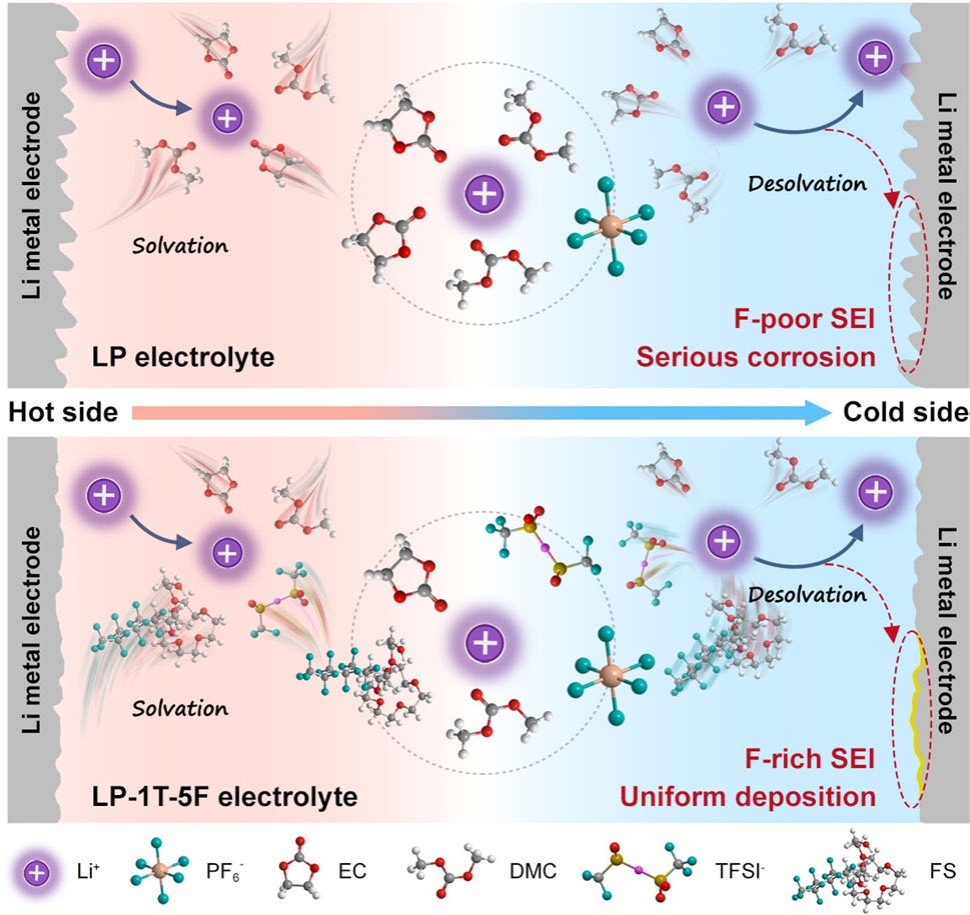
Schematic diagram of ions in LP (pristine) electrolyte and LP-1T-5F electrolyte

Herein, we typically proposed a dual-salt electrolyte with fluorosurfactant (FS) additive to yield a high output power and energy density of LTECs, simultaneously. Specifically, the dual-anion aggregated sheath can be formed by incorporating hexafluorophosphate (PF_6_^−^) with bistrifluoromethanesulfonimide (TFSI^−^) anions. The addition of FS enables an anion-rich solvated structure and significantly facilitates the Li^+^ transfer kinetics in electrolyte, which accelerates the separation of anions and cations. As illustrated in Fig. 1, the FS additive is beneficial for the formation of F-rich solid-state electrolyte interphase (SEI) on the Li metal electrode, which could hinder the corrosion of metal electrode compared with F-poor SEI using pristine electrolyte during ions deposition. Benefitting from such optimized Li^+^ solvation sheath and stable electrolyte/electrode interface, a high power density and remarkable thermopower as well as good durability can be obtained by as-fabricated device. Such proposed device demonstrates an ultrafast response ability and extends in the application of heat-to-electricity conversion, inspiring potential for development of high-performance LTECs using in practical applications.

## Experimental Section

### Materials

LiPF_6_ salts and EC-DMC (EC:DMC = 1:1 vol%) solvent were purchased from Shanshan Advanced Materials (Quzhou) Co., Ltd. LiTFSI salts (LT) were purchased from Zhangjiagang Guotai Huarong New Chemical Co., Ltd. Capstone FS-3100 (FS) was purchased from Chemours Chemicals Co., Ltd. Li foil (100 μm) was provided by China Energy Lithium Co., Ltd. Graphite was purchased from Jiangxi Zhengtuo New Energy Technology Co., Ltd.

### Preparation of Electrolytes

Typically, the basis electrolyte (LP) was 1.0 mol L^−1^ LiPF_6_ electrolyte, which prepared using LiPF_6_ salt and EC-DMC solvent based on the molar ratio. LP-*x*T (*x*: the weight ratio of LT) electrolyte was prepared by adding a certain amount of LT. Moreover, the FS was introduced into the LP-*x*T electrolyte, and denoted as LP-*x*T-*y*F (*y*: the weight ratio of FS). All the electrolytes were prepared and stored in an argon-filled glovebox (oxygen < 0.01 ppm, water < 0.01 ppm) at room temperature.

### Material Characterization

Raman spectroscopy analyses were conducted on HORIBA Scientific LabRAM HR Evolution with 532 nm laser. The ^19^F and ^7^Li nuclear magnetic resonance (NMR) spectra were recorded on 600 MHz Bruker NMR spectrometer at ambient temperature and used DMSO-D_2_O as an internal standard in a coaxial NMR tube. Morphology of plated Li metal was observed by field emission scanning electron microscopy (FE-SEM, Tescan Lyra3 gmu). X-ray photoelectron spectroscopy (XPS) measurements of surface on Li metal were acquired by Thermo Scientific K-Alpha + with monochromatized Al Kα radiation. It should be mentioning that the surface of Li metal was sputtered using a monatomic Ar^+^ beam with an ion energy of 4000 eV and a raster size of 2.0 mm, and an estimated sputter rate of 0.36 nm s^−1^ calibrated for Ta_2_O_5_. Ionic conductivity of electrolyte was measured by S230 SevenCompact conductivity meter. Thermal conductivity of electrolyte was calculated based on the steady-state method, while that for electrode was tested by Hot Disk TPS 2500S instrument. The specific surface area values of samples were measured by ASAP 2460 aperture analyzer (micromeritics) using argon.

### Performance Measurement of the LTECs

The diffusion curves were recorded by chronoamperometry (CA) method under overpotential of 10 mV. Electrochemical impedance spectroscopy (EIS) was implemented within a range of 10^6^ to 10^−2^ Hz. All the thermoelectrochemical performances were evaluated on a standard electrochemical workstation (CHI 760e) with non-isothermal H cell using designed electrolyte. The temperature difference between cold side and hot side was generated by using temperature-controlled water baths. To evaluate the properties of electrolytes in symmetric LTECs, Li metal foil was used as the working electrode and counter/reference electrode without any processing. For asymmetric LG-LTECs, the graphite electrode (mass loading: ~ 1 mg cm^−2^), containing graphite powder, acetylene black and poly(vinylidene fluoride) (PVDF) with a mass ratio of 8: 1: 1 on carbon coated copper foil, was employed as cathode on hot side, while Li foil was used as anode on cold side.

## Results and Discussion

### Thermoelectric Properties of Electrolytes

Typically, the thermovoltage generated by applying temperature difference in LTEC can be derived from two effects, including the thermodiffusion effect of ions in electrolytes and the thermogalvanic effect of Li^+^ in electrodes. The voltage contributed by thermodiffusion is related to the concentration difference between anions and cations during mass transport and the change of Eastman entropy, while the thermovoltage induced by the thermogalvanic effect is associated with the redox reactions together with desolvation process between ions and electrodes in LTECs [12, 30]. Both of them are inseparable from the feature of the electrolyte ions and the solvation structure of cations [23]. As a demo, the Li^+^ presents in form of solvated Li^+^ in electrolyte by interacting with surrounding solvent molecules or anions. It is facile to enhance the thermovoltage of LTEC via the solvation engineering. Thus, LiPF_6_ (LP) and LiTFSI (LT) are reasonably employed to formulate dual-salt electrolyte in ethylene carbonate (EC) and dimethyl carbonate (DMC) solvents, which is further modified by FS additive. The thermoelectrochemical behaviors of each electrolyte tested by H-type LTECs with symmetrical Li metal electrodes under various temperature differences are displayed in Fig. S1. Notably, the output voltage of each system increases gradually and follow with a slight voltage drop, which can be ascribed to the overpotential leading by the stripping of Li [31, 32]. Finally, the output voltage reaches stability as the temperature incrementally increased. Comparing to the output voltages of LTEC based on LP basic electrolyte under different temperature differences, such values are significantly enhanced after introducing LT salt and FS additive, demonstrating the efficiency of electrolyte additives for the improvement of thermoelectrochemical sensitivity. In general, the Seebeck coefficient/thermopower (*S*_*i*_) fitted by voltage difference (∆*V*) and temperature difference (∆*T*) is applied to determine the corresponding thermoelectrochemical behavior of LTECs [33]. As shown in Figs. S2 and S3, the LTEC based on LP electrolyte holds a relatively low *S*_i_ value of 0.89 mV K^−1^. Compared with the LP electrolyte, the ionic conductivity of LP-*x*T (*x* represents the weight ratio of LT) electrolytes is slightly reduced from 10.73 to 10.42 mS cm^−1^, but the thermoelectrochemical behavior of LP-*x*T is obviously improved due to the introduction of anion (TFSI^−^) with relatively large size, especially for the LP-1T electrolyte [34, 35]. When adding a certain amount of LT into LP electrolyte, the maximum *S*_i_ value can be improved to 1.08 mV K^−1^. It should mention that the free ions in LP-*x*T electrolyte are easier to form ion pairs with the increasing LT content to a relatively high level (1.5 and 2.0 wt%) [36]. As a result, the *S*_i_ value is decreased to 0.95 mV K^−1^ using LP-2T electrolyte. Under this case, the LP-1T electrolyte is optimized for the further thermoelectrochemical measurements of LTECs due to its relatively high *S*_i_ value and moderate ionic conductivity.

**Fig. 2:**
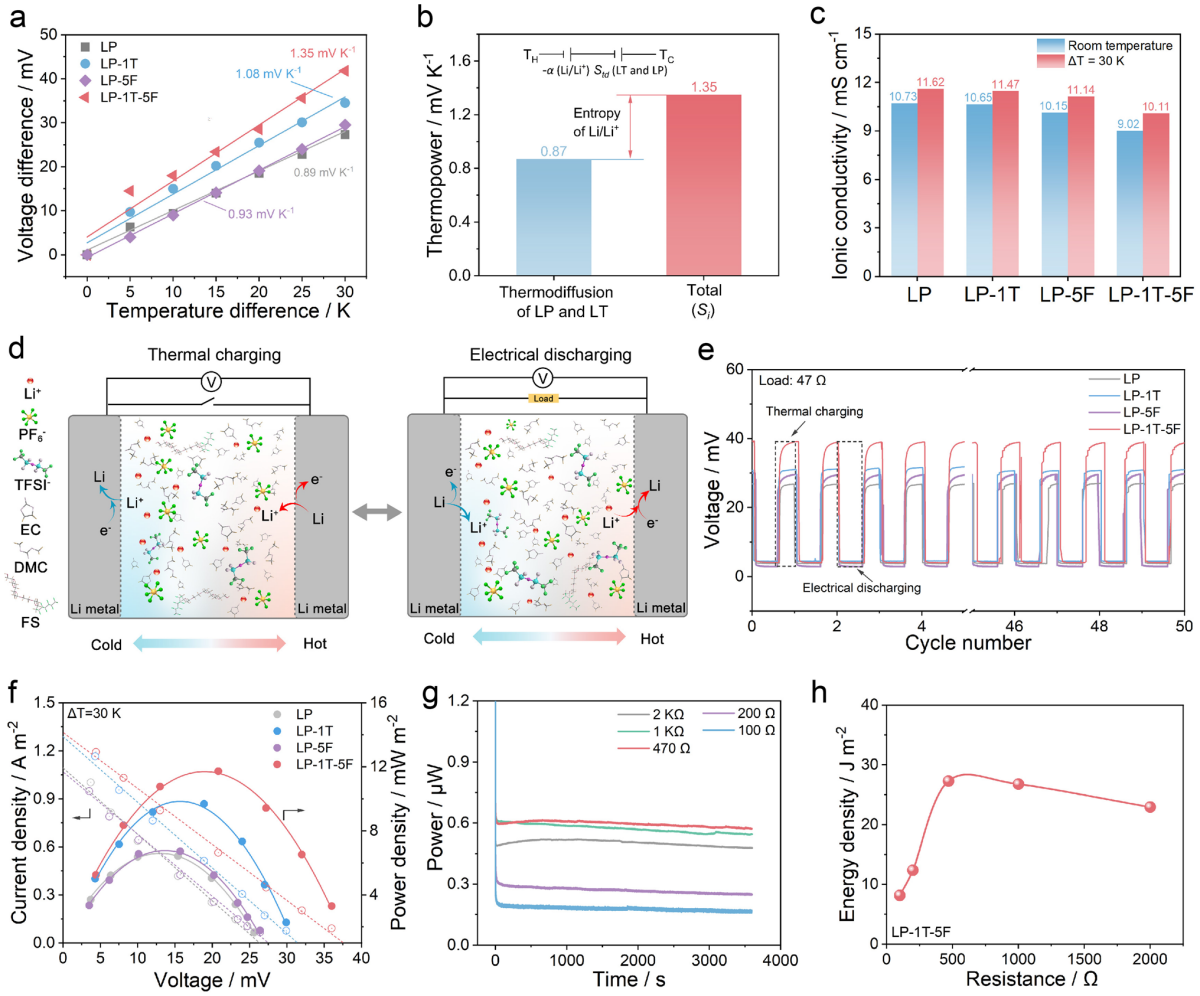
Thermally charging properties of LTECs. **a** The voltage difference of LP, LP-1T, LP-5F, and LP-1T-5F at different values of ∆*T*. **b** Thermopower caused by the thermodiffusion and the corresponding proportion of the total thermopower. **c** Ionic conductivity under room temperature and 328.15 K. **d** Schematic illustration of LTECs in thermal charging process and electrical discharging process. **e** Voltage curve of thermal charging/load discharging process at a temperature difference of 30 K. **f** Current densities and power densities under various voltages. **g** Power change under continuous discharge operation with different external resistors at 30 K and **h** the corresponding energy densities

In addition, solvent in electrolyte, which plays a crucially important role in Li^+^ solvation, has been known to significantly affect the performance of heat to electricity [12]. To achieve the efficient enhancement of thermopower, functional additives are employed to modify the solvation environment of Li^+^. As guidelines, the promising additives could not only participate in the Li^+^ solvation process, but also exhibit low desolvation energy [30]. FS as a commercial fluorosurfactant demonstrates attractive promise in the improvement of diffusion and desolvation of Li^+^ owing to the unique interaction between Li^+^ and ethylene oxide groups (-CH_2_CH_2_O-) in FS [37, 38]. To investigate the effect of added FS on heat-to-electricity conversion, a series of LP-1T-*y*F (*y* represents the weight ratio of FS) electrolytes assisted by FS additive based on LP-1T are performed for LTECs. As summarized in Figs. S4 and S5, the highest *S*_i_ value of 1.35 mV K^−1^ can be achieved by such LTEC using LP-1T-5F electrolyte. With the content of FS further increasing, the thermopower is slightly decreased; nevertheless, it is still higher than that of LTEC without the addition of FS. This phenomenon may associate with the ethylene oxide unit in FS, which favorably interacts with Li^+^ and becomes the reversible Li–O bonds during solvation process, thereby increasing the entropy change of system [39], while the trend of ionic conductivity is opposite to the thermopower, which continues to decrease with the addition of FS. It can be ascribed to the concentration of ionizable lithium salt which begins to decrease with the increasing proportion of FS in designed electrolyte, resulting in the decrease in ion carrier concentration [37]. Meanwhile, the thermopower of LP-5F without LT was also characterized with the value of 0.93 mV K^−1^ (Fig. S6), which is higher than LP-based LTEC but less than the LP-1T-5F-based LTEC, because part of thermopower is contributed by the solvent-dependent entropy change between ions and solvents, which is associated with the interactions of ions and their solvation shells. Consequently, the thermopower of LTEC based on LP-1T-5F is significantly enhanced compared with LTECs using LP, LP-1T and LP-5F respectively (Fig. 2a). Moreover, the thermopower contribution from the thermodiffusion effect is determined by employing symmetrical stainless steel (SS) electrodes instead of Li metal, yielding a value of 0.87 mV K^−1^ (Figs. 2b and S7a). Accordingly, the relative contribution of LP-1T-5F-based LTEC to overall thermopower is calculated as 64.4% of the thermodiffusion effect contribution and 35.6% of the redox entropy change from Li/Li (Fig. S7b). As shown in Fig. 2c, different components have a great impact on the thermal sensitivity of electrolytes. At room temperature (298.3 K), the LP electrolyte demonstrates the highest ionic conductivity of 10.73 mS cm^−1^. LP-1T-5F electrolyte shows a relatively low ionic conductivity of 9.02 mS cm^−1^, suggesting ~ 84.1% of the ionic conductivity of the LP electrolyte. It is worth mentioning that the ionic conductivity of LP-1T-5F electrolyte is significantly improved to 10.11 mS cm^−1^ at 328.3 K (∆T = 30 K), indicating the highest increase ratio of 112% to the value at room temperature. These results further confirm the high sensitivity of LP-1T-5F electrolyte to temperature. Moreover, the effective thermal conductivity (λ) of various electrolytes is measured by a steady-state method based on the law of conservation of heat flow. During experiments, the temperature distribution on electrolyte is determined by Fourier infrared thermal imager. As shown in Fig. S8, the LP-1T-5F electrolyte displays relatively lower heat conduction than those of LP, LP-1T and LP-5F electrolytes under the same conditions. As a result, a thermal conductivity of 0.61 W m^−1^ K^−1^ can be calculated for the LP-1T-5F electrolyte, lower than that of LP (0.87 W m^−1^ K^−1^), LP-1T (0.71 W m^−1^ K^−1^), and LP-5F (0.67 W m^−1^ K^−1^) electrolyte, respectively (Fig. S9). When comparing to the LP electrolyte, the decrease in thermal conductivity of LP-1T, LP-5F, and LP-1T-5F electrolytes may be greatly attributed to the slightly increase in electrolyte viscosity (from 121.3 to 147.7 cP) with the addition of LT and FS (Fig. S10). Specifically, the effective thermal conductivity in LTEC is comprised of thermal conduction and convection, and the increase in electrolyte viscosity significantly suppresses heat convection [40, 41].

Apart from the high thermopower, the study of power density and energy density as well as quasicontinuous working mode in LTECs system also is crucial to evaluate the conversion efficiency of available electrical energy from low-grade heat. As illustrated in Fig. 2d, charges were accumulated on the hot side during the stripping of Li^+^ from Li electrode along with the heat flux-induced ion diffusion from hot side to cold side. During the electrical discharging process, charge transfer from hot side to cold side can be realized by connecting an external resistor. Meanwhile, the plated Li^+^ in cold side would be stripped and re-plated in hot side, which confirms the rechargeability of LTECs for heat-to-current conversion by adopting the temperature gradient. Such mechanism during thermal charging and electrical discharging process was further confirmed by an non-isothermal three-electrode system using Li metal as working/reference/counter electrode (Fig. S11). Accordingly, the heat energy could be converted into electricity continuously. As demonstration, LP, LP-1T, LP-5F, and LP-1T-5F electrolyte-based LTEC can obtain a near-saturated output voltage about 26, 32, 28, and 40 mV after thermal charging at the temperature difference of 30 K, respectively (Fig. 2e). The thermovoltage decayed rapidly and approached to ~ 0 V within 10 s via loading an external resistance of 47 Ω at the temperature difference of 30 K. It is worth mentioning that the voltage can recover back within 10 s with adopting the temperature gradient after disconnecting the loading resistor. Under this case, the consumed species were resupplied by the diffusion of ions. The transfer of electrons during open-circuit condition can be carried out by removing between electrode and electrolyte interface or switching between non electronic state and the electronic state in the chemicals. Moreover, the quasicontinuous charge–discharge curves of LTECs using LP-1T-5F electrolyte over 50 cycles indicate the satisfying thermoelectrochemical stability for the continuous conversion of low-grade heat (Fig. 2e), which is much comparable to that of LP, LP-1T, and LP-5F electrolyte-based LTEC. It is worth mentioning that the lifespan of LTECs highly depends on the durability and stripping/plating efficiency of Li metal with different electrolytes. Comparing with the symmetric Li//Li cell using LP electrolyte, the cell using LP-1T-5F electrolyte exhibits relatively stable and low voltage polarization even after stripping/plating for 1000 h at the same conditions (Fig. S12a). Moreover, the performances of asymmetric Li//Cu cells in Fig. S12b indicate that the optimized LP-1T-5F electrolyte can endow the stripping/plating of Li metal electrodes with satisfying average Coulombic efficiency (~ 86.3%). However, the Li//Cu cell using the LP electrolyte displays a slightly low average efficiency of ~ 85.5%, which may be caused by some irreversible reactions occurred in electrode/electrolyte interfaces. Such results further confirm that the introduction of LT and FS into typical LP electrolyte is beneficial for achieving stable and durable Li metal-based devices. As profiled in Fig. 2f, the current density (*I*) and the power density (*P*) of LTECs are evaluated by using a series of fixed resistors (*R*) based on $I=\frac{V}{R}$ and $P=\frac{{V}^{2}}{R}$, respectively. Among them, the LP-1T-5F electrolyte-based LTEC exhibits the highest short-circuit current density of 1.3 A m^−2^. Notably, the parabolic power density–voltage curve of LTECs using LP-1T-5F electrolyte demonstrates the maximum power density of 11.55 mW m^−2^, which is much better than that of LTECs using LP (6.41 mW m^−2^), LP-1T (9.68 mW m^−2^), and LP-5F (6.72 mW m^−2^) electrolyte, respectively. Detailly, the power change curves of LTEC based on LP-1T-5F electrolyte with different loading resistances (100–2000 Ω) are summarized in Fig. 2g. The related power of LTEC is dramatically decayed with the decrement of thermovoltage in the initial state, which can be attributed to the discharge process with connecting the external circuit. As the discharge proceeds, a new steady-state can be eventually achieved. Furthermore, the converted energy during thermoelectrochemical processes with various loading resistances can be determined by integrating output power within 1 h. Consequently, a comparable energy density of 27.26 J m^−2^ can be obtained from LTEC using LP-1T-5F electrolyte with the load of 470 Ω (Fig. 2h), indicating the possibility and durability of LTEC in the harvesting of low-grade heat.

### Solvent–Solute Interaction in Designed Electrolytes

**Fig. 3:**
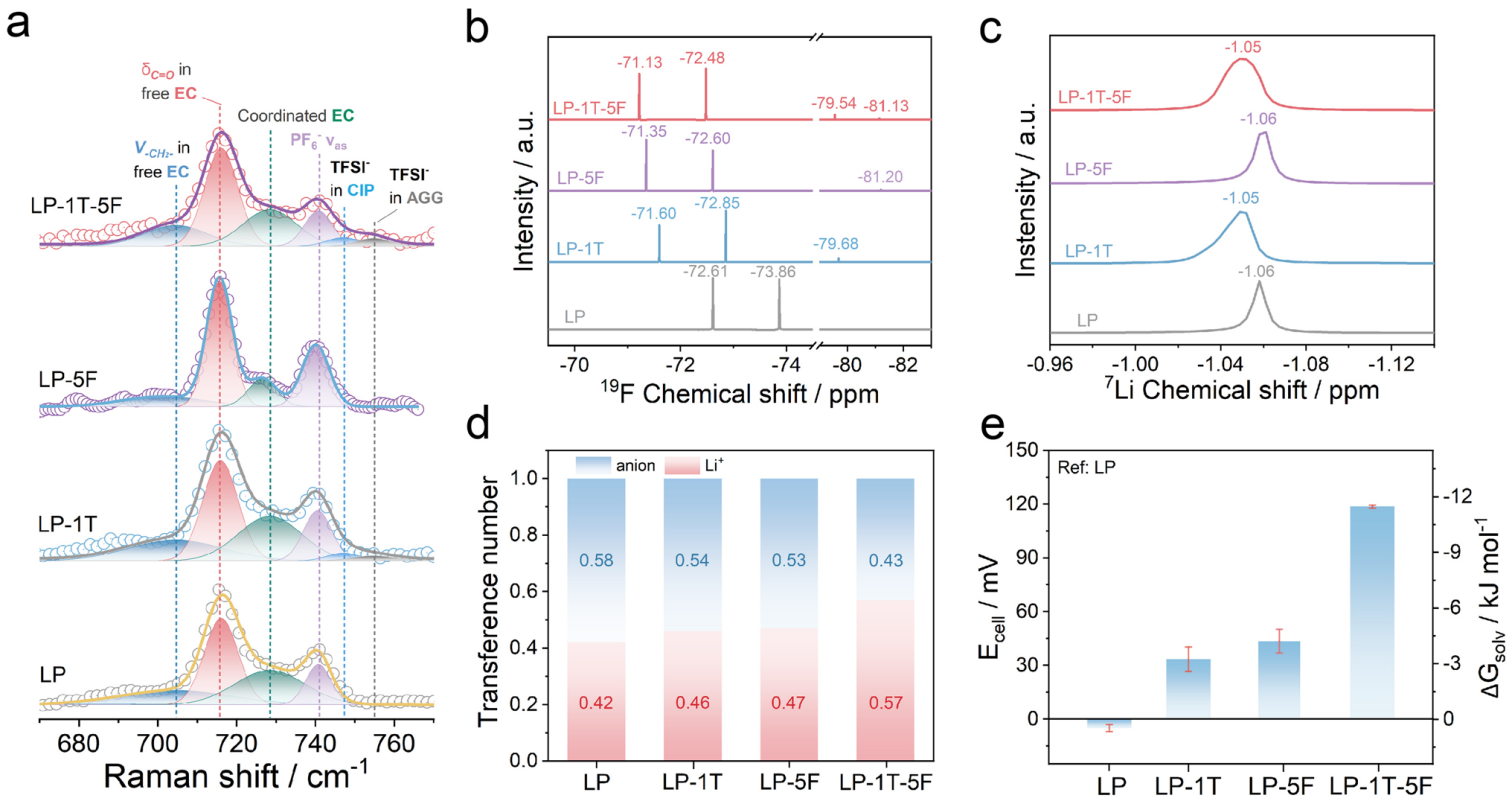
Coordination solvation chemistry for electrolytes. **a** Raman spectra, **b** ^19^F NMR spectra, and **c** ^7^Li NMR spectra for LP, LP-1T, LP-5F, and LP-1T-5F. **d** Li^+^ and anions transference number of electrolyte. **e** Measured cell potentials at different electrolytes and the corresponding solvation energy values

The enhanced thermoelectrochemical performances of LP-1T-5F-based LTEC are possibly determined by the modification of pristine properties of electrolytes such as solvation structure and ion diffusion coefficient. Therefore, we conducted Raman spectra and NMR spectra as powerful techniques to understand the possible relationship between electrolyte solvation structure and thermoelectrochemical performances [42]. As shown in Figs. 3a and S13, several characteristic bands of Raman spectra can be observed in as-designed electrolytes. Detailly, the Raman spectrum of pure EC-DMC solvent delivers typical bands at 704, 717, 892, and 917 cm^−1^, which can be corresponding to the −CH_2_− rocking mode of EC, C=O breathing of EC, the ring breathing mode of EC and CH_3_−O stretching vibration of DMC, respectively [43, 44]. Compared to EC-DMC solvent, four additional bands (728, 741, 901 and 935 cm^−1^) appear in the spectrum of LP, LP-1T, LP-5F, and LP-1T-5F electrolyte, which can be assigned to the coordinated EC, A_1g_ asymmetric vibrations of PF_6_^–^ (ν_P–F_), the coordination cluster of EC and DMC molecule with Li^+^, respectively [45–47]. Generally, the interactions between Li^+^ and anions in electrolytes may form three solvation structures: solvent separated ion pair (SSIP), contact ion pairs (CIP) and aggregate solvates (AGG), respectively [48]. The formed CIP/AGG modes in LP-1T and LP-1T-5F indicate the TFSI^–^ interacts with Li^+^, further confirming by the Raman spectra in Fig. 3a. Besides, the dissociation peak at 742 cm^−1^ is overwhelmed by the ν_P–F_ band of PF_6_^–^, suggesting a dual-anion (PF_6_^–^ and TFSI^–^)-aggregated Li^+^ solvation structure together with coordination of EC and DMC in LP-1T and LP-1T-5F electrolytes [49, 50]. ^19^F and ^7^Li NMR spectra were measured to evaluate ion binding environment in different electrolytes using DMSO-D_2_O as the internal standard to correct the chemical shift. As shown in Fig. 3b, the ^19^F NMR spectrum for LP can be attributed to the PF_6_^–^ (− 72.61 and − 73.86 ppm) [51]. With the introducing of LT in LP electrolyte, the ^19^F NMR spectra for LP-1T and LP-1T-5F can be divided into two parts, including the F atoms in the PF_6_^–^ (around − 71 and − 73 ppm) and the F atoms in TFSI^–^ groups (around − 79 ppm). A new peak (around − 81 ppm) detected in the LP-5F and LP-1T-5F electrolytes can be corresponding to the end groups (–CF_3_) in perfluorinated chain of FS. It is worth mentioning that the chemical shift to a lower field in LP-1T, LP-5F, and LP-1T-5F indicates the decreased electron density around the F atom in PF_6_^–^ by the introduced deshielding effect. The interaction between anions and solvent molecules in the solvation structure not only affects the electron density, but also causes the chemical shift. Figure 3c displays the ^7^Li NMR spectra of as-used electrolytes. Due to the strong electron-withdrawing groups in TFSI^–^ groups deprived electrons around Li^+^, the ^7^Li signal of LP-1T and LP-1T-5F displays a downfield shift compared to that of LP and LP-5F systems [52]. Based on these results, the induced aggregate cluster in LP-1T-5F electrolyte immobilize PF_6_^–^ and TFSI^–^ anions and boost the Li^+^ transport, which further explains the attractive thermoelectrochemical performances of LP-1T-5F-based LTEC.

To elucidate the significant influence of such Li^+^ solvation structure on Li^+^ diffusion, the Li^+^ transference number (t_Li_^+^) on behalf of the relative ionic diffusivity in electrolyte was conducted. The value of t_Li_^+^ is determined by electrochemical impedance spectrum (EIS) and chronoamperometry (CA) measurement applying a positive overpotential of 10 mV in the Li symmetric cell. Figure S14 shows the corresponding EIS spectra and CA curves of LP, LP-1T, LP-5F and LP-1T-5F systems. In conclusion, the LP-1T-5F electrolyte with the t_Li_^+^ value of 0.57, which is larger than LP (0.42), LP-1T (0.46), and LP-5F (0.47) (Fig. 3d), can be attributed to segmental structural motion in place of vehicular motion and the low desolvation energy of FS additive. More specifically, the diffusion of Li^+^ with its tightly bound solvated shells as one entity, typically known as vehicular motion, is dominated ionic diffusion mechanism in most commercial liquid electrolyte such as LP, leading to a sluggish diffusion owing to the plump sheath of Li^+^ [53]. When solvated Li^+^ in LP-1T and LP-1T-5F is under AGG mode, it diffuses via successive ion dissociation/association exchange across different solvation shells, in which the diffusion is known as structural motion with an enhanced Li^+^ mobility [53]. To gain a deeper insight into energetics of Li^+^ solvation, the solvation energy was applied to analyze. The Li^+^ solvation energy in electrolyte was probed by potentiometric method based on the relationship $\Delta G=-zEF$, where *z*, *F* and *E* are electrons transferred number, Faraday constant and electrical potential. To our knowledge, the electromotive force (EMF) stems from the difference of free energy between two different electrodes in the traditional batteries. Meanwhile, the EMF can be generated by the free energy difference of Li^+^ solvation in two different electrolytes with the same electrodes on both sides. Accordingly, the LP-1T, LP-5F and LP-1T-5F were measured against a reference electrolyte of LP at room temperature. As shown in Fig. 3e, LP-1T have significantly more positive cell potential (33.4 mV) and more negative solvation energy (− 3.2 kJ mol^–1^) comparing with LP (− 5 mV, 0.48 kJ mol^–1^). It can be inferred that the TFSI^–^ participates in the solvation shell of LP-1T electrolyte, where such incorporation can reduce the desolvation energy of Li^+^ [54]. Moreover, the addition of FS in LP-5F electrolyte leads to a larger difference in solvation energy, which can be ascribed to the effect of fluorination in FS [55]. The combined function of LT and FS and the difference in solvation energy of LP-1T-5F electrolyte are obviously enhanced.

**Fig. 4:**
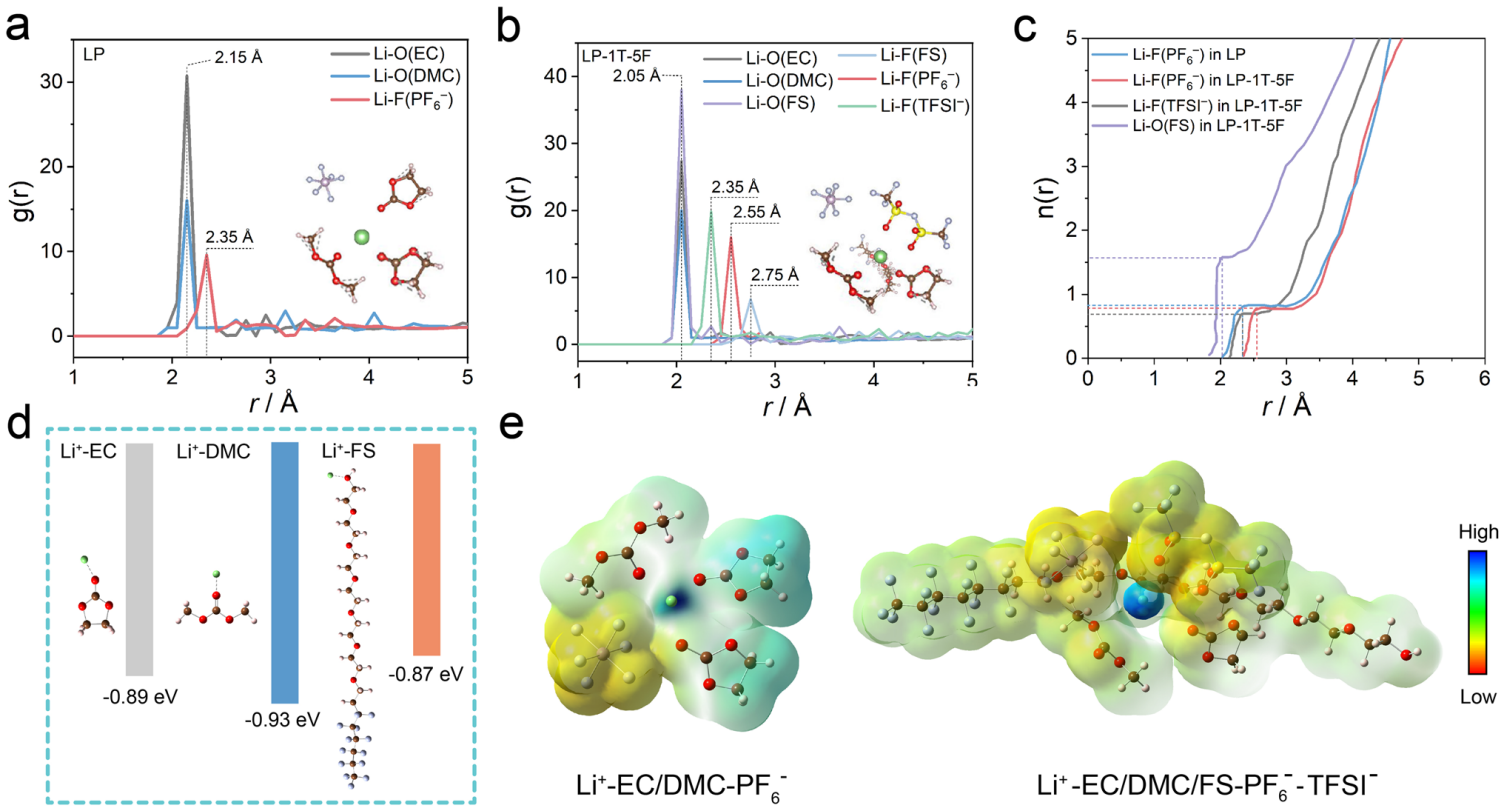
RDFs of **a** LP and **b** LP-1T-5F electrolyte. **c** n(r) of Li^+^-anions/FS in LP and LP-1T-5F electrolyte from RDFs. **d** Binding energy between Li^+^ and solvent/additive. **e** ESP of solvation structure in the LP and LP-1T-5F electrolyte

To further investigate the effect of the interaction between ions and its solvation shells on thermoelectrochemical performance, the coordination structures of solvents and ions were quantitatively studied by classic molecular dynamic (MD) simulations. The representative snapshots and localized Li^+^ solvation structures for LP and LP-1T-5F are shown in Fig. S15a, b. The average interaction environment of Li^+^ was described by radial distribution function (RDF) and the corresponding coordination number (Fig. 4a, b). According to the RDF results of LP electrolyte, a significantly higher intensity of Li–O peak from EC and DMC than LiF peak from PF_6_^–^ can be observed in the solvation structure of LP electrolyte (Fig. 4a), suggesting a inner solvation sheath dominated by EC and DMC. This implies that the organic species are preferentially formed on the surface of electrode, which is unfavorable for the stability of electrode/electrolyte interfaces. Interestingly, the distance corresponding to the peaks of EC and DMC toward the barycenter of Li^+^ has slightly changed after the incorporation of TFSI^–^ and FS, whereas LiF peak of PF_6_^–^ shifts to 2.55 Å and the LiF peak of TFSI^–^ appears at 2.35 Å, indicating the participation of TFSI^–^ into the first solvation shell of Li^+^ (Fig. 4b). Moreover, the cumulative numbers of FS, PF_6_^–^ and TFSI^–^ anions with Li^+^ are 1.56, 0.79 and 0.70 in LP-1T-5F, while that of PF_6_^–^ anions with Li^+^ is 0.81 in LP (Fig. 4c). These results indicate the capability of TFSI^–^ anions and FS for regulating the solvation structure of Li^+^. Besides, the binding energy of Li^+^-FS (− 0.87 eV) is lower than that of Li^+^-EC (− 0.89 eV) and Li^+^-DMC (–0.93 eV) (Fig. 4d). The weak interaction of Li^+^-FS can be regulated easier than the strong interaction of Li^+^ with surrounding solvents, which would be beneficial for the desolvation process and cause a relatively large entropy change in the system [30, 56]. The charge distribution around Li^+^-solvation structures and electronic surface potential (ESP) charges of LP and LP-1T-5F electrolytes are shown in Fig. 4e. Comparing with additive-free LP electrolyte, the negative charge distribution of the solvation structure in LP-1T-5F electrolyte mainly distributes on PF_6_^–^ and DMC, indicating the difference in internal driving force for the formation of solvent–solute clusters. Based on these results, it can be concluded that the Li^+^ solvation structure in LP-1T-5F electrolytes is dominated by PF_6_^–^, TFSI^–^, and FS, together with the partial coordination of EC and DMC solvent molecules, in line with the findings from the Raman results.

### Electrode–Electrolyte Interfacial Analysis

**Fig. 5:**
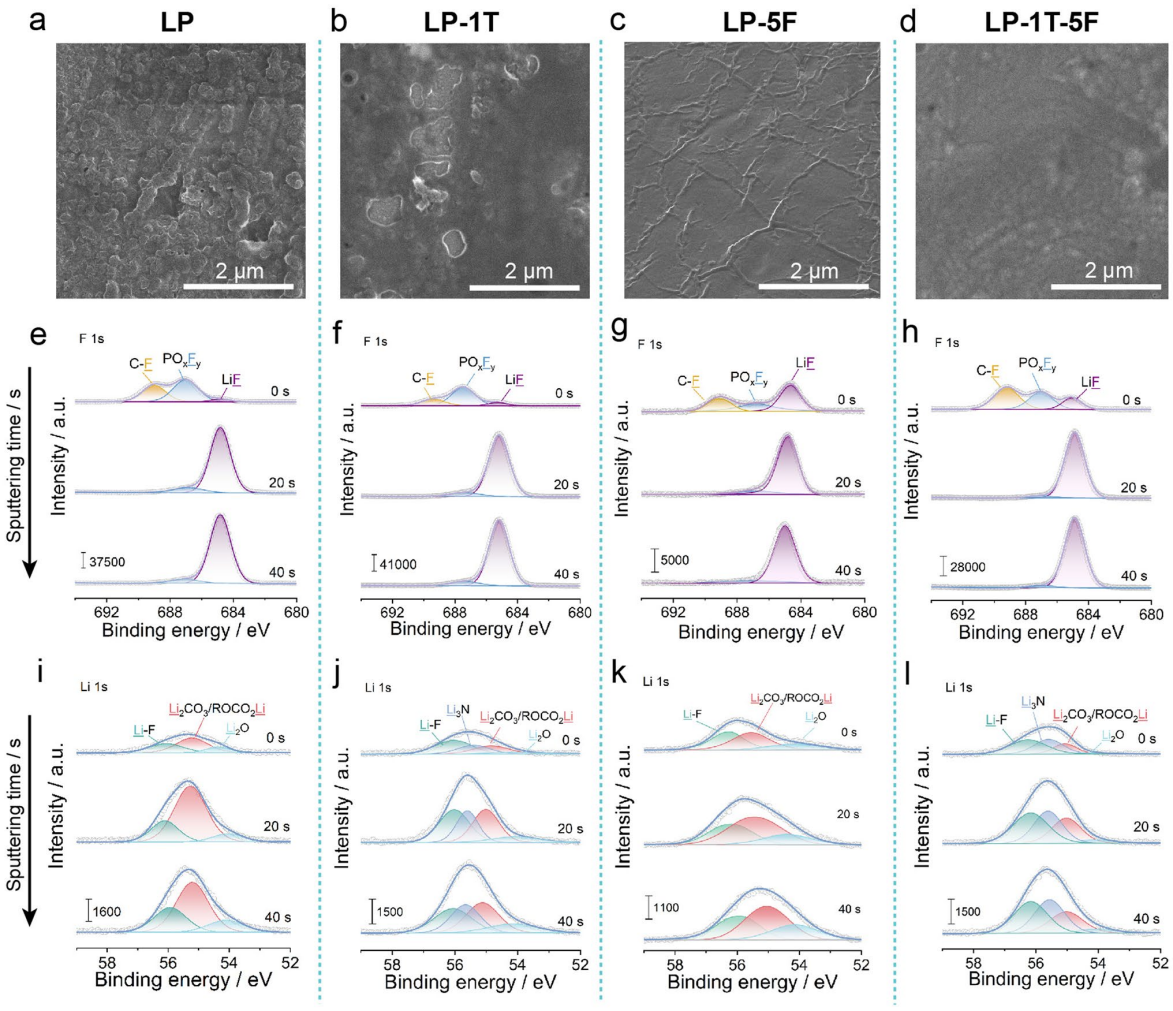
Characterization of the interfacial compositions of cycled Li electrodes on the cold side. SEM images of Li metal in **a** LP, **b** LP-1T, **c** LP-5F, and **d** LP-1T-5F electrolytes after charging, respectively. XPS spectra of **e–h** F 1*s* and **i-l** Li 1*s* for Li metal surface with increasing etching depth

The morphologies of Li metal electrode engineered by as-designed electrolytes after thermoelectrochemical tests are presented in Fig. 5a–d. Clearly, the mossy and nodule-like Li deposition morphology can be observed in LP and LP-1T electrolyte after cycling (Fig. 5a, b). Of note, the Li electrode in LP-5F and LP-1T-5F electrolyte explicitly depicts a relatively uniform and smooth surface with the assistance of FS (Fig. 5c, d), manifesting that the addition of FS can induce homogeneous deposition behavior of Li^+^ on the surface of Li metal and suppress the uncontrolled formation of Li dendrites. Furthermore, the composition of Li metal electrode/electrolyte interface was elucidated by in-depth XPS. In the F 1*s* spectra, the detected peak at 688.8, 686.7, and 684.5 eV can be ascribed to the C–F, PO_*x*_F_*y*_, and LiF, which are the most common constituents of the SEI (Fig. 5e–h) [54, 57]. Because of the abundant F functional groups in the FS, the higher amount of LiF is presented in the SEI formed in LP-5F and LP-1T-5F-contained electrolyte, which is beneficial for the interface stability [57]. However, the low conductivity of LiF may lead to the poor diffusion kinetics of Li^+^. Besides, the component ratios of LiF species in such four electrolytes all increase obviously with etching, while the C-F peak disappears and the content of PO_*x*_F_*y*_ peak notably diminishes. The Li 1*s* spectra detected in SEI layer with LP, LP-1T, LP-5F and LP-1T-5F electrolytes all are included three peaks of LiF (56.1 eV), Li_2_CO_3_/RCOO_2_Li (55.0 eV), and Li_2_O (54.1 eV) (Fig. 5i–l). With the increase in etching time, the content of Li_2_O in these four electrolytes all gradually increases. Interestingly, a new peak at 55.6 eV assigned to Li_3_N species (main byproduct of the TFSI^−^ decomposition) can be observed in the SEI layer formed by LP-1T and LP-1T-5F electrolyte, which is beneficial for the transport of Li^+^. Briefly, the FS modified dual-anion-aggregated Li^+^ solvation shell can generate a LiF/Li_3_N-rich heterostructured SEI layer with stable characteristic and low ions diffusion energy barrier, thereby enabling the less side-reaction on electrode and boosting the cycling durability of LTECs.

### Proof-of-Concept Asymmetric LTECs Device

**Fig. 6:**
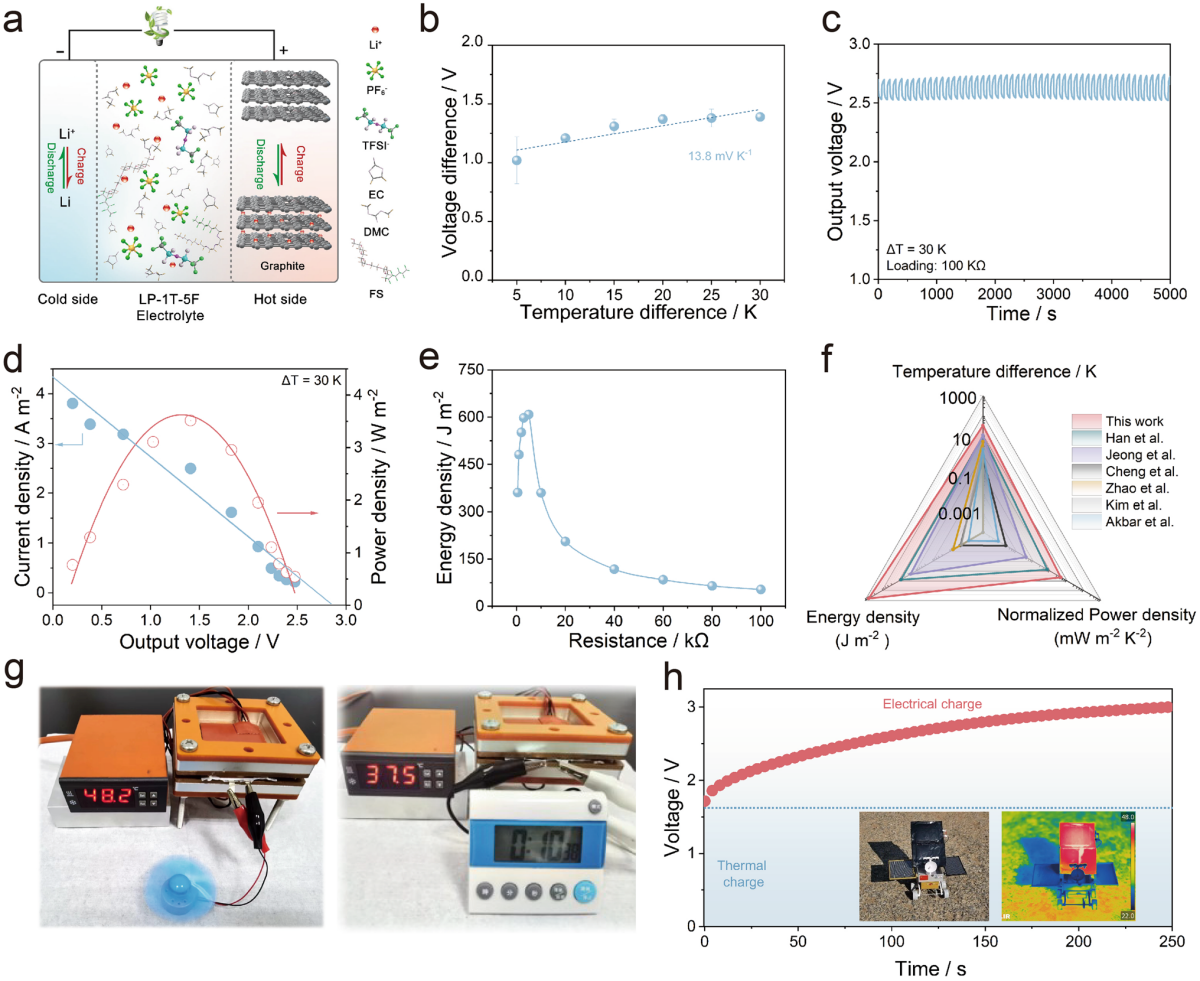
**a** Schematic drawing of the LG-LTECs (LiǀLP-1T-5FǀGraphite). **b** Thermopower of LG-LTECs. **c** The measured voltage under continuous-working mode. **d** Current density and power density versus voltage. **e** Energy density with different external resistors. **f** Performance comparison of the temperature difference, energy density, and normalized power density with previous results in literatures. **g** Fan and timer powered by the device. **h** Power charging curve of LG-LTECs after thermal charging process (insets display the photographs of LG-LTECs modules placed in outdoor sunshine and the corresponding temperature distribution detected by the infrared imaging system)

To further evaluate the possibility of as-optimized LP-1T-5F electrolyte in the practical construction of lithium-based full cells, we developed an asymmetric LTEC (LG-LTEC) using Li metal anode in cold side, graphite cathode in hot side, and LP-1T-5F electrolyte. It should be mentioning that the graphite electrode exhibits relatively lower thermal conductivity (56.6 W m^−1^ K^−1^) and higher specific surface area (4.2 m^2^ g^−1^) than those of Li metal (62.0 W m^−1^ K^−1^, 2.9 m^2^ g^−1^), implying enhanced performances of as-developed LTECs (Figs. S16 and S17) [58]. Before testing, the graphite electrode was pre-intercalated by Li^+^ ions using the galvanostatic discharge technique until the voltage of LG-LTEC device reached 0.01 V. As illustrated in Fig. 6a, the pre-intercalated Li^+^ ions in graphite electrode would be extracted at the hot side and diffused to the surface of Li metal at the cold side for immediate deposition, in which the energy conversion and storage can be simultaneously implemented in the graphite electrode. As shown in Fig. S18, the thermally charging behavior of LG-LTEC was measured with the temperature difference from 5 to 30 K. Notably, the output voltage of LG-LTEC can reach up to 1.42, 2.04, 2.48, 2.59, 2.70, and 2.77 V with thermal charging for thirty minutes at the temperature difference of 5, 10, 15, 20, 25, and 30 K, respectively. In fact, such output voltage is contributed by the thermovoltage from heat-to-current conversion and the electrochemically self-charging process caused by the concentration polarization and ohmic polarization in the LTEC [59, 60]. In detail, the ion diffusion in the electrolyte is unable to keep up with the ion concentration change near the electrode caused by the redox reaction, which lead to a concentration gradient near the electrode and voltage recovery. Figure S18 profiles the electrochemically self-charging curves without temperature gradient. After deducting this contribution, a relatively high thermovoltage of 1.26 V can be retained by LG-LTEC. As fitted in Fig. 6b, the LG-LTEC achieved a comparable thermopower value of 13.8 mV K^−1^ among reported heat-to-electricity devices [20, 40, 61, 62]. It is obvious that such thermopower value is much more competitive than that of LTECs using symmetrical Li metal electrodes and LP-1T-5F electrolytes, which can be ascribed to various types of electrodes with different working mechanisms during thermally charging process. In detail, the LG-LTEC involves hybrid mechanisms between the thermogalvanic effect and the thermodiffusion effect of Li^+^ (Fig. S19), which can be further divided into several elementary processes: (i) electron transport in composite electrodes, (ii) Li^+^ diffusion inside active materials, (iii) Li^+^ transfer at active material/electrolyte interfaces, and (iv) Li^+^ diffusion in electrolyte. Consequently, the proportion of thermodiffusion for ions in electrolyte and the graphite electrode is 6.3% and 73.3%, respectively. The proportion of thermogalvanic processes for graphite/lithiated-graphite (G/Li_*x*_G) and Li/Li^+^ is 16.9% and 3.5%, respectively. The corresponding long-term cyclic power generation of LG-LTEC was performed with a fixed load resistor of 100 kΩ, as shown in Fig. 6c. Impressively, LG-LTEC exhibits a stable voltage of 2.70 V even over 50 cycles, demonstrating the low energy decay and satisfying stability of thermally rechargeable behavior. It worth mentioning that the LG-LTEC can thermally recharge after the load discharging, and the thermal charging process makes little influence on the subsequent galvanostatic charging process (Fig. S20). In addition, the output power densities of LG-LTEC have been carried out with various load resistors of 0.47–100 kΩ (Fig. 6d). Significantly, the LG-LTEC generates a short-circuit current of 4.33 A m^−2^ and the maximum power density of 3.59 W m^−2^ at a temperature difference of 30 K. As shown in Fig. S21, voltage and output power reached a steady-state operation mode after a few seconds with the saturation of external resistors. Accordingly, the output energy of LG-LTEC can be calculated by integrating power density-time curves based on the relationship of $E=\int P {\text{d}}t$, delivering energy up to 607.96 J m^−2^ with the resistor of 5 kΩ (Fig. 6e). Moreover, LG-LTEC exhibits a high *P*_max_/(Δ*T*)^2^ of 3.99 mW m^−2^ K^−2^ at Δ*T* = 30 K, which are both the key parameters for evaluating the capability of heat to electricity. Such values catch up with the higher level among the LTECs ever reported (Fig. 6f and Table S1) [1, 63–66].

To demonstrate the application of LG-LTECs in case of reality, a module by connecting two pouch cells of LG-LTECs in series was developed, which can directly power various small electronic device such as fan and smart timer (Fig. 6g). Moreover, collecting solar heat and converting into electricity whether on earth or in space are the attractive potential application and adapt to global energy revolution. As a proof of concept, a prototype integrating solar absorber with energy conversion, consisting of 2 units LG-LTECs, was constructed, where the back of LTECs can provide suitable operating temperature through attached solar thermal absorbing film. The feasibility for solar heat-to-electricity conversion by LG-LTECs was conducted in Nanjing during the summertime as shown in the inset in Fig. 6h. Impressively, the designed prototype can work normally after one hour of shining, and recharge from thermal-charge (about 1.6 V) to 3.0 V (Fig. 6h), demonstrating the availability and long-term operation of developed LG-LTECs.

## Conclusions

In summary, the dual-anion ester electrolyte with fluorosurfactant additive is reasonably developed for high-performance LTECs based on entropy increase effect. Briefly, the Li^+^ solvation sheath can be controllably adjusted by the introduction of the anions and FS, which forms a dual-anion-aggregated Li^+^ solvation structure as revealed by theoretical simulations and spectroscopies. Benefitting from the advantages of such coordination structure, the kinetics and thermoelectrochemical performances of Li-based LTECs can be significantly promoted using LP-1T-5F electrolyte, which delivers a high thermopower of 1.35 mV K^−1^. By combining the functional graphite electrode with Li metal electrode, as-developed asymmetric LTECs exhibit further improved thermopower of 13.8 mV K^−1^ and *P*_max_/(Δ*T*)^2^ of 3.99 mW m^−2^ K^−2^ as well as a remarkable energy density of 607.96 J m^−2^. Such device provides a feasible approach for harnessing low-grade heat and converting into electricity. This work introduces the concept of fluorosurfactant additive to regulate Li^+^ solvation and optimize the electrode/electrolyte interface, which has profound implications for the construction of promising devices in thermal energy-harvesting applications.

## Supplementary Information

Below is the link to the electronic supplementary material.

Supplementary file1 (PDF 1242 KB)
