# Supplementary material for: Solvation Engineering via Fluorosurfactant Additive Toward Boosted Lithium-Ion Thermoelectrochemical Cells
Source: Nanomicro Lett. 2024 Jan 4;16:72. doi: 10.1007/s40820-023-01292-2 (PMC10766582; doi:10.1007/s40820-023-01292-2)
Supplement: Supplementary file 1 — Supplementary file1 (PDF 1242 KB) [file 40820_2023_1292_MOESM1_ESM.pdf]

Supporting Information for

Solvation Engineering via Fluorosurfactant Additive Toward Boosted Lithium-Ion Thermoelectrochemical Cells

Yinghong Xu[1], Zhiwei Li[1], Langyuan Wu[1], Hui Dou[1], Xiaogang Zhang[1,]*

[1]Jiangsu Key Laboratory of Electrochemical Energy Storage Technologies, College of Material Science and Technology, Nanjing University of Aeronautics and Astronautics, Nanjing 211106, P. R. China

*Corresponding author. E-mail: azhangxg@nuaa.edu.cn (Xiaogang Zhang)

S1 Computational Methods

Molecular dynamic (MD) simulations were performed via Groningen Machine for Chemical Simulations (GROMACS) to study the Li^+ solvation structure. The object system was modeled by $LiPF_6$, LT, FS, EC, DMC molecules stacked in supercell. The simulated system is periodic with a unit cell size of 3.4404 nm × 3.4404 nm × 3.4404 nm and $\alpha=\beta=\gamma=90^o$. The OPLS force field, which is suitable for electrolyte solution, was used to optimize sample structures for preliminary structural optimization. Atomic charges of ions were multiplied by scale factor 0.73 to correct the polarization effect of anion and cation. The Ewald summation method with an accuracy of 0.002 kcal/mol was used to calculate long-range electrostatic potentials. The velocity Verlet algorithm with a time step of 1 fs was used to integrate the motion equations under an NVT ensemble with the Nose–Hoover thermostat at 300 K. During each simulation, the system was first equilibrated for 1 ns, followed by 10 ns of production steps. All simulations were repeated for five times with random initial velocities and the average results of these five runs were reported. Next, MD simulations were further carried out for 20 ns with a time step of 1 fs per integration step under the ensemble conditions of NVT (300 K). System energy can be obtained through structural optimization using the energy minimization.

The binding energy and electronic surface potential were conducted using density functional theory (DFT) method. The structures of the studied complexes (denoted by Li^+-EC, Li^+-DMC, Li^+-FS) were fully optimized at the B3LYP-D3BJ/def2-SVP level of theory. The solvent effect (50%EC+50%DMC) was included in the calculations using the solvation model based on the density (SMD) model. The vibrational frequencies of the optimized structures were carried out at the same level. The structures were characterized as a local energy minimum on the potential energy surface by verifying that all the vibrational frequencies were real. The calculation formulas of binding energy (E_b) are shown below: $E_b(A\text{-}B)=E(AB)-E(A)-E(B)$, in which AB represent the complexes of Li^+-EC, Li^+-DMC, Li^+-FS.

S2 Supplementary Figures and Tables

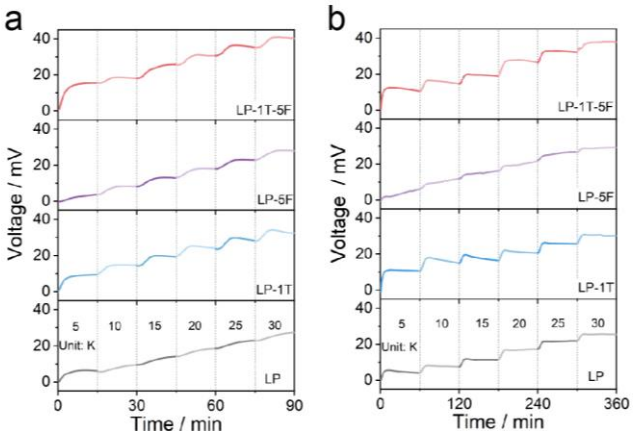

Fig. S1 Evolution of the output voltage with varied temperature gradients under (**a**) 90 min and (**b**) 360 min

As shown in Fig. S1a and b, the output voltages of LP, LP-1T, LP-5F and LP-1T-5F with a temperature difference of 30 K over 360 min are 25.5, 30.3, 29.0 and 38.0 mV, respectively. In fact, such obtained values are comparable to those of LP (27.0 mV), LP-1T (32.4 mV), LP-5F (28.2 mV), and LP-1T-5F (40.1 mV) after charging over 90 min. Besides, we used a voltage varying rate <0.3 mV min^{-1} to distinguish the near-saturation status. It can be found that LTECs needs about 15-20 min to reach the near-saturation status during the thermal charging process. Thus, we have applied 15 min as testing time to evaluate the heat-to-current behavior of as-constructed LTECs in this work.

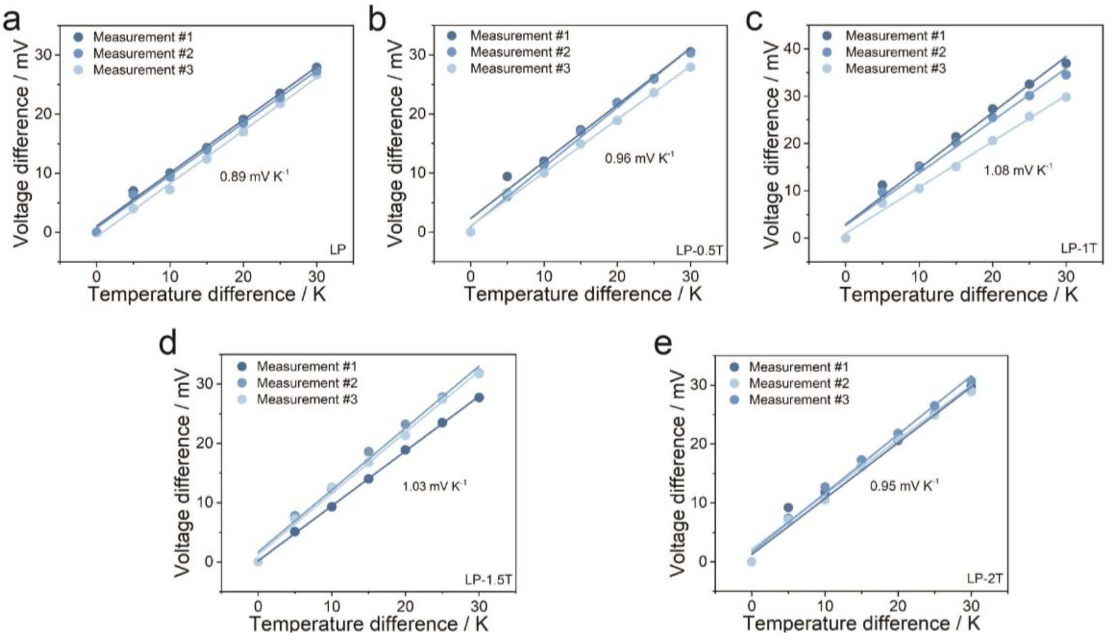

Fig. S2 The fitting results of voltage difference versus different values of ΔT for (**a**) LP, (**b**) LP-0.5T, (**c**) LP-1T, (**d**) LP-1.5T, (**e**) LP-2T. [LP-*x*T, x is content of LT.]

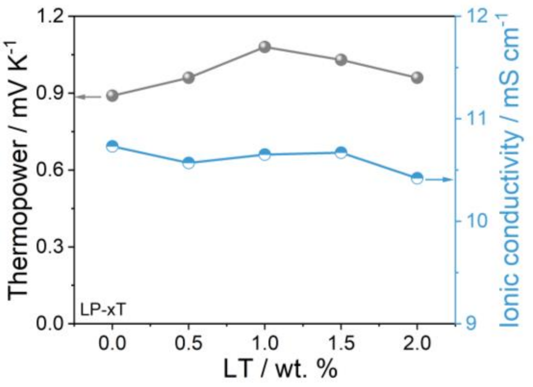

Fig. S3 The ionic conductivity and thermopower of LP-*x*T with different amounts of LT

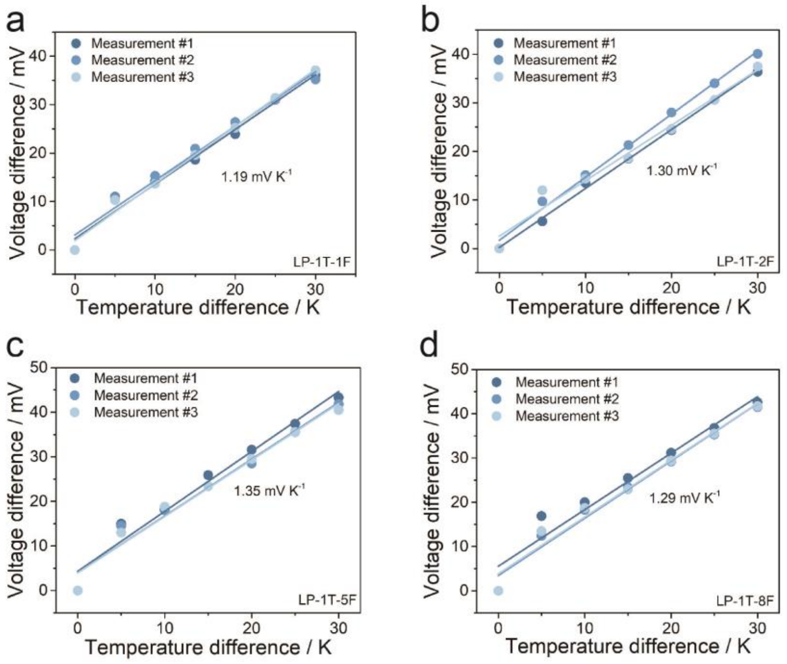

Fig. S4 The fitting results of voltage difference versus different values of ΔT for (**a**) LP-1T-1F, (**b**) LP-1T-2F, (**c**) LP-1T-5F, (**d**) LP-1T-8F. [LP-1T-*y*F, y is content of FS.]

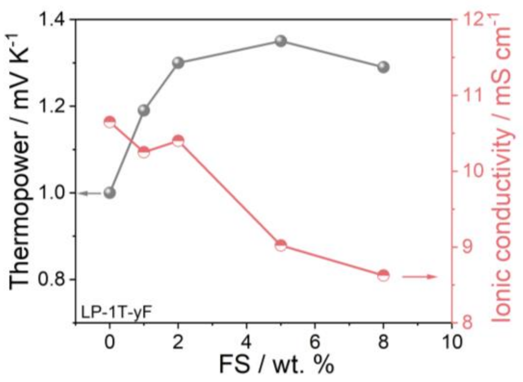

Fig. S5 The ionic conductivity and thermopower of LP-1T-*y*F with different amounts of FS

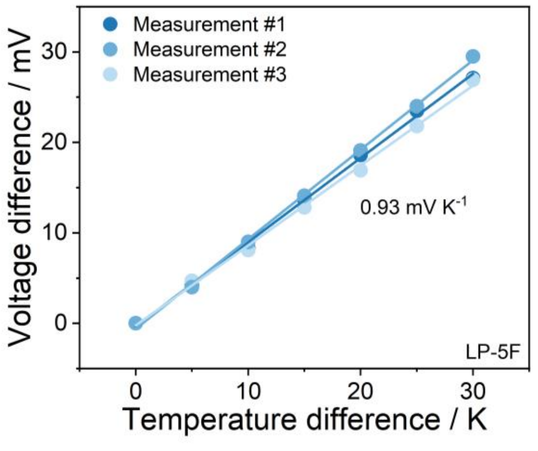

Fig. S6 The thermopower of LP-5F

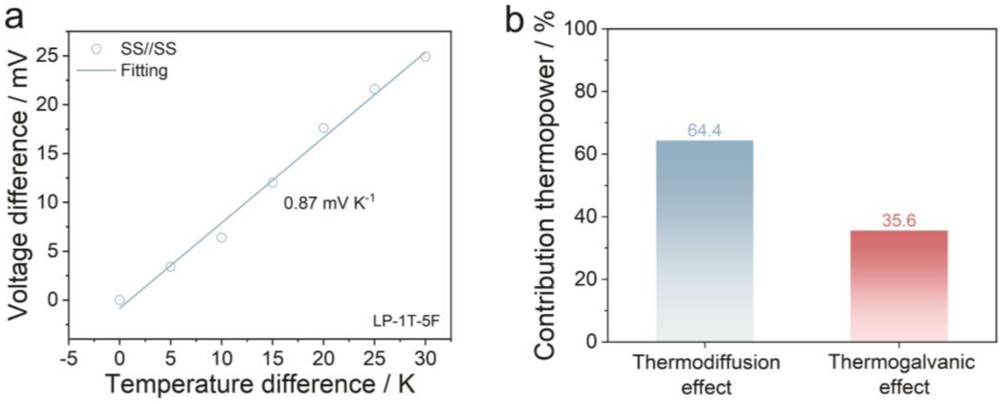

Fig. S7 (**a**) The voltage difference of stainless steel SS|LP-1T-5F|SS LTEC at different value of temperature difference. (**b**) Fractional contribution to thermopower of LP-1T-5F based LTEC

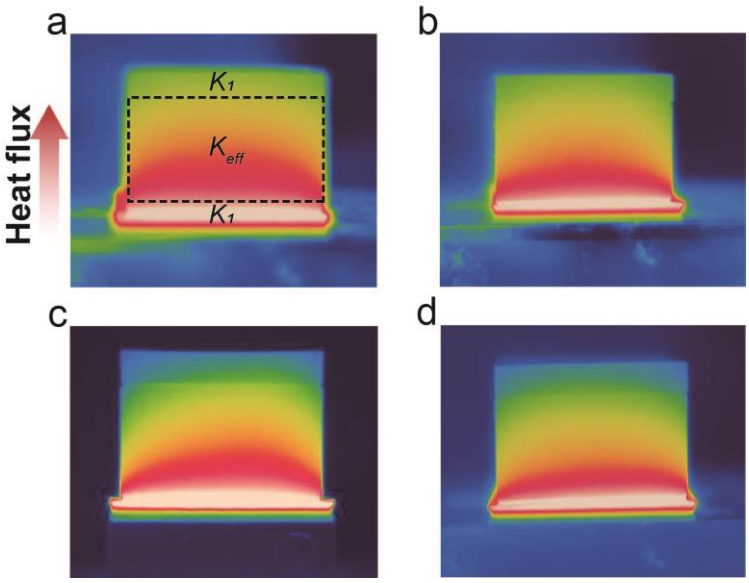

Fig. S8 The infrared images of (**a**) LP, (**b**) LP-1T, (**c**) LP-5F, and (**d**) LP-1T-5F electrolyte under same heat input after one hour

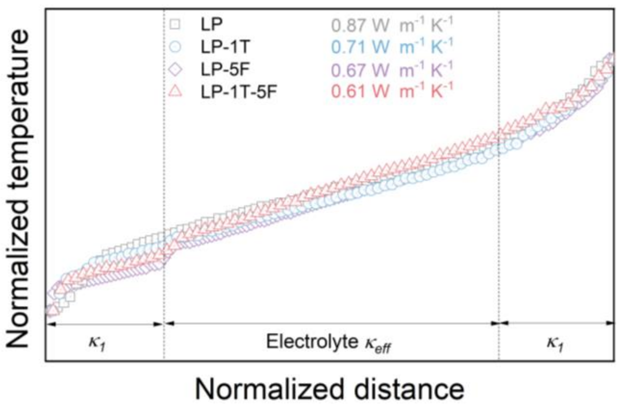

Fig. S9 The normalized temperature curves of LP, LP-1T, LP-5F, and LP-1T-5F electrolyte based on the infrared imagery

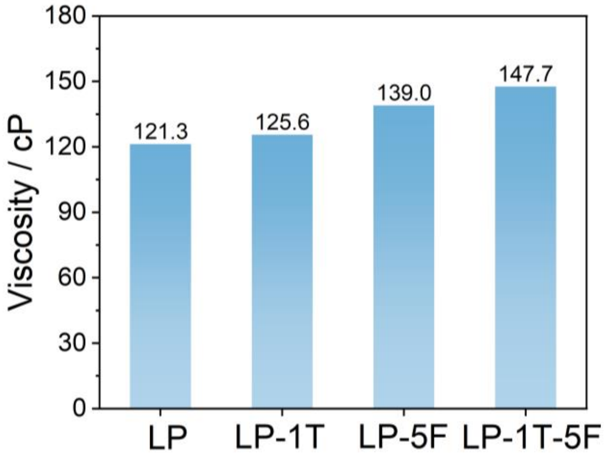

Fig. S10 The viscosities of designed electrolytes

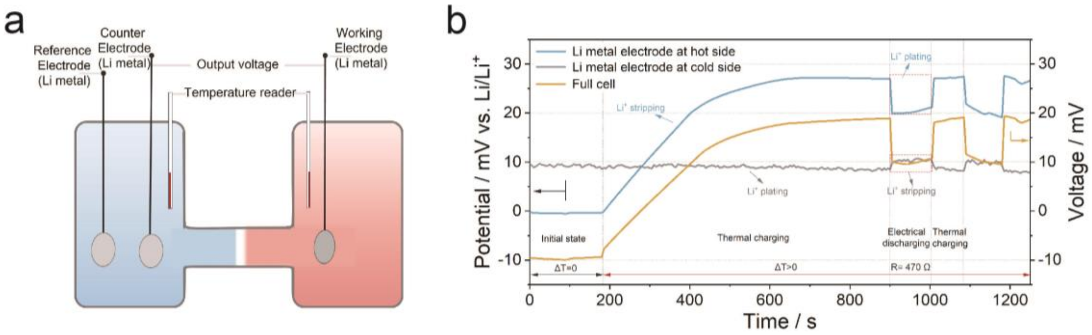

Fig. S11 (**a**) Non-isothermal system of LP-1T-5F based LTEC for measuring the potential change of each electrode during thermal charging and electrical discharging process. The work electrode (WE) was lithium metal, whereas lithium metal was also used as the reference electrode (RE) and counter electrode (CE). (**b**) The corresponding real-time investigation of potential for each electrode and the voltage of full cell

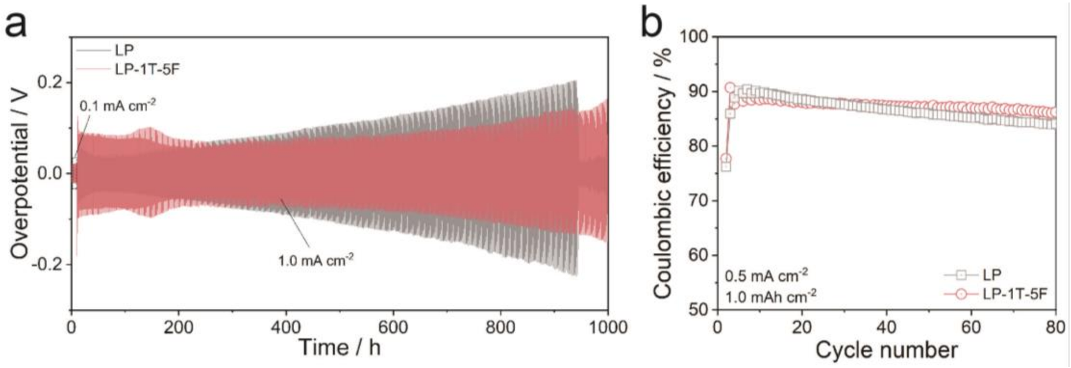

Fig. S12 (**a**) The cycling stability of Li//Li symmetric cells, and (**b**) stripping/plating efficiency of Li//Cu asymmetric cell using LP and LP-1T-5F electrolytes

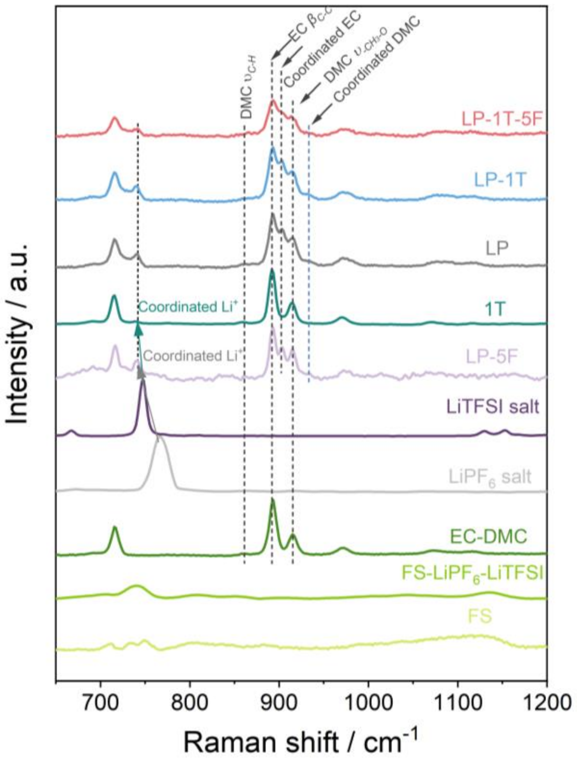

Fig. S13 Raman spectra of designed electrolytes, lithium salts and solvents

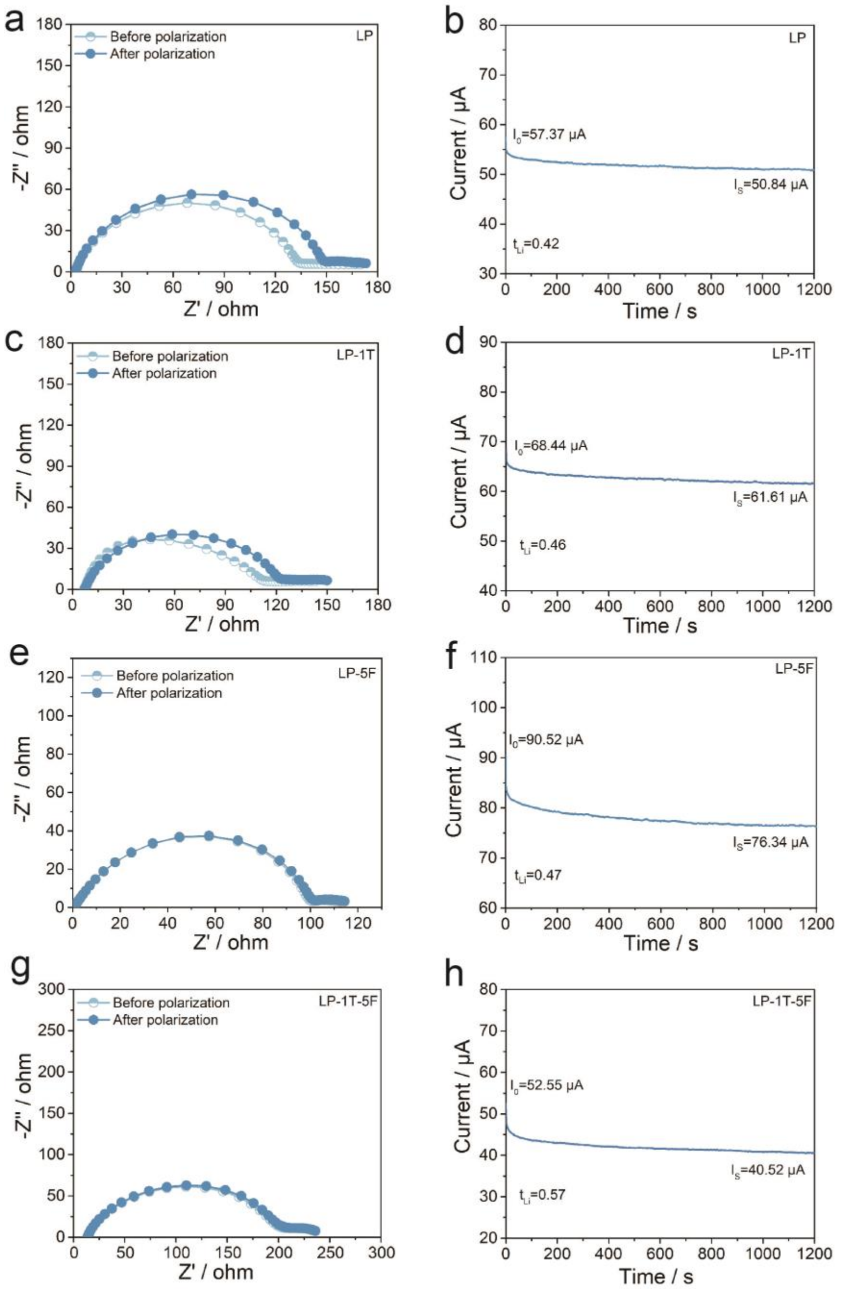

Fig. S14 (**a, c, e, g**) Electrochemical impedance spectroscopy (EIS) diagram before and after the polarization in designed electrolytes, and (**b, d, f, h**) polarization results at a positive overpotential of 10 mV

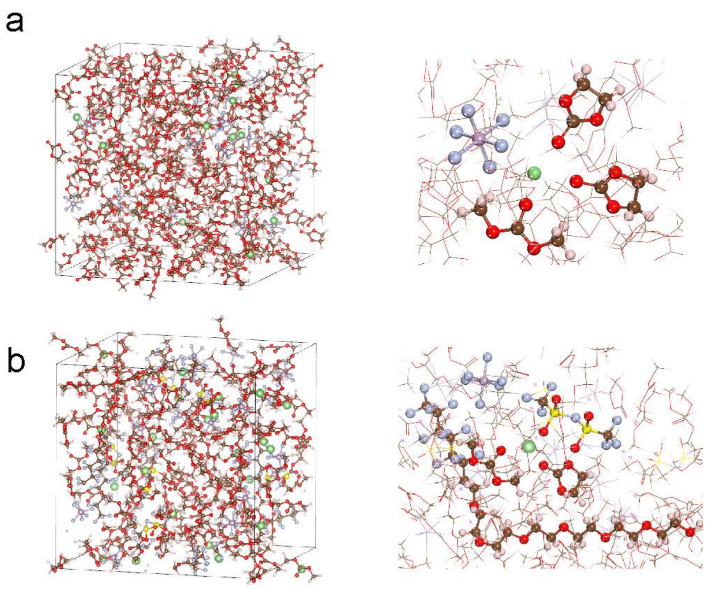

Fig. S15 Snapshots of (**a**) LP, (**b**) LP-1T-5F solvation structure from MD simulations

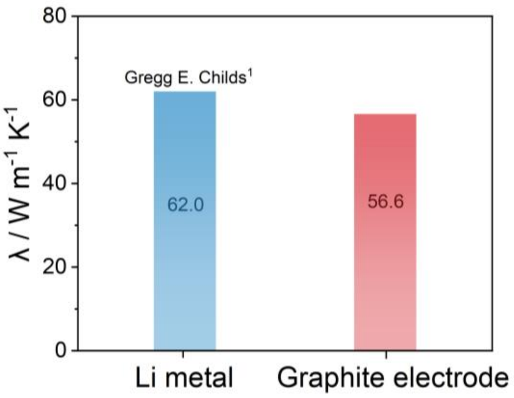

Fig. S16 The thermal conductivity of Li metal and graphite electrodes

The value of Li metal is reported by Gregg E. Childs[1] and that of graphite electrode is measured by the hot disk method.

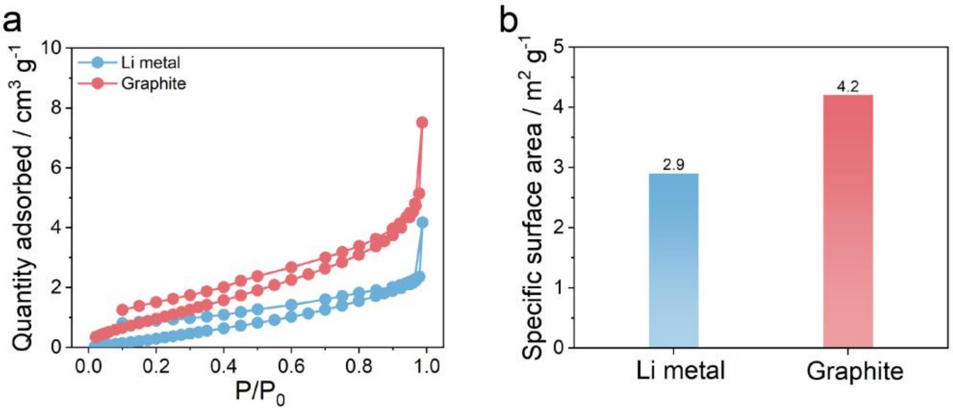

Fig. S17 (**a**) Ar adsorption-desorption isotherms, and (**b**) corresponding specific surface area values for various materials

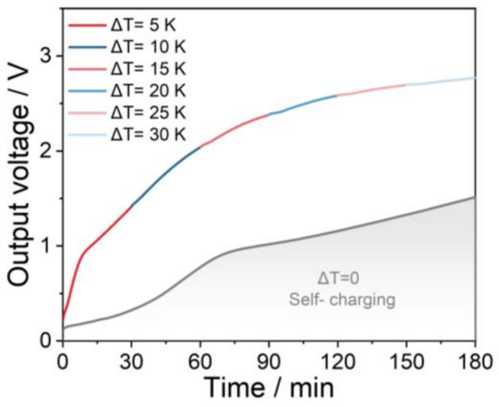

Fig. S18 Electrochemically self-charging behavior of LG-LTECs and output voltage of the LG-LTECs at different ΔT

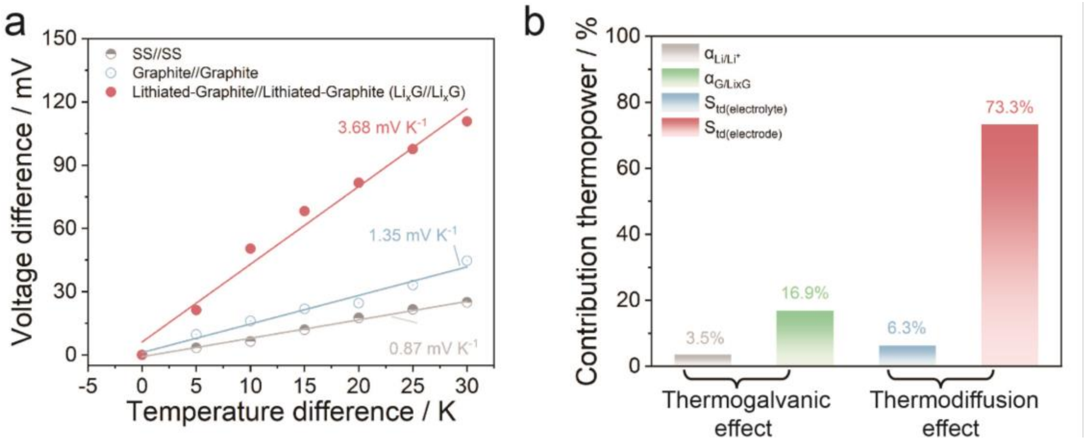

Fig. S19 (**a**) Thermopower of LP-1T-5F based electrolyte with different electrode and (**b**) fractional contribution to thermopower of LG-LTECs

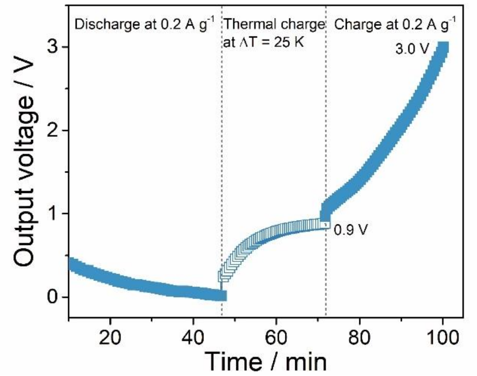

Fig. S20 Charge/discharge behavior of LG-LTECs under galvanostatic and/or thermal hybrid modes

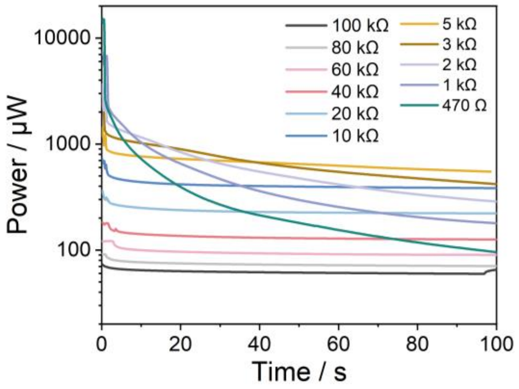

Fig. S21 Output power with different external resistors under ΔT=30 K

Table S1 Summary of various electrolyte of reported in the literatures

Electrolyte	Electrode	S_e (mV K^{-1})	P (W m^{-2})	E (J m^{-2})	Refs.
LP-1T-5F	Li metal//Graphite	13.8	3.59	607.96	This work
LP-1T-5F	Li metal//Li metal	1.35	0.0115	27.26	This work
PhNP/T-PhNP	Cu//Cu	26.5	0.0208	8.33	S2
Gelatin-KCl-$FeCN^{4-/3-}$	Cu|Au//Cu|Au	17.0	0.042	12.8	S3
PANI-PAAMPSA-PA	Ag//Ag	8.1	2.38×10^{-6}	7.14×10^{-4}	S4
PVDF-HFP-EMIM:DCA	SWNT//SWNT	26.1	0.84×10^{-6}	2.23×10^{-4}	S5
PEO-NaOH	CNT|Au//CNT|Au	10	/	6×10^{-3}	S6
PSS-H-GO	Metal//Metal	12.6	/	2	S7
PPP-SiO_2	SWCNT//SWCNT	14.8	1.8×10^{-4}	0.25	S8

Supplementary References

[S1] G. Childs, L. Ericks, R. Powell, NBS Monograph, 1976, 131.

[S2] C. Chi, M. An, X. Qi, Y. Li, R. Zhang et al., Selectively tuning ionic thermopower in all-solid-state flexible polymer composites for thermal sensing. Nat. Commun., 2022, 13, 221. https://doi.org/10.1038/s41467-021-27885-2

[S3] C.-G. Han, X. Qian, Q. Li, B. Deng, Y. Zhu et al., Giant thermopower of ionic gelatin near room temperature. Science, 2020, 368, 1091. https://doi.org/10.1126/science.aaz5045

[S4] Z. A. Akbar, J.-W. Jeon, S.-Y. Jang, Intrinsically self-healable, stretchable thermoelectric materials with a large ionic Seebeck effect. Energy Environ. Sci. 2020, 13, 2915-2923. https://doi.org/10.1039/C9EE03861B

[S5] H. Cheng, X. He, Z. Fan, J. Ouyang, Flexible quasi-solid state ionogels with remarkable seebeck coefficient and high thermoelectric properties. Adv. Energy Mater., 2019, 9, 1901085. https://doi.org/10.1002/aenm.201901085

[S6] D. Zhao, H. Wang, Z. U. Khan, J. C. Chen, R. Gabrielsson et al., Ionic thermoelectric supercapacitors. Energy Environ. Sci., 2016, 9, 1450-1457. https://doi.org/10.1039/C6EE00121A

[S7] M. Jeong, J. Noh, M. Z. Islam, K. Kim, A. Sohn et al., Embedding aligned graphene oxides in polyelectrolytes to facilitate thermo-diffusion of protons for high ionic thermoelectric figure-of-merit. Adv. Funct. Mater., 2021, 31, 2011016. https://doi.org/10.1002/adfm.202011016

[S8] X. He, H. Cheng, S. Yue, J. Ouyang, Quasi-solid state nanoparticle/(ionic liquid) gels with significantly high ionic thermoelectric properties. J. Mater. Chem. A, 2020, 8, 10813-10821. https://doi.org/10.1039/D0TA04100A
